# Alternative methods of processing bio-feedstocks in formulated consumer product design

**DOI:** 10.3389/fchem.2014.00026

**Published:** 2014-05-13

**Authors:** Nicolai Peremezhney, Philipp-Maximilian Jacob, Alexei Lapkin

**Affiliations:** Department of Chemical Engineering and Biotechnology, University of CambridgeCambridge, UK

**Keywords:** processing bio-feedstocks, expert systems, evolution-based product design

## Abstract

In this work new methods of processing bio-feedstocks in the formulated consumer products industry are discussed. Our current approach to formulated products design is based on heuristic knowledge of formulators that allows selecting individual compounds from a library of available materials with known properties. We speculate that most of the compounds (or functions) that make up the product to be designed can potentially be obtained from a few bio-sources. In this case, it may be possible to design a sequence of transformations required to convert feedstocks into products with desired properties, analogous to a metabolic pathway of a complex organism. We conceptualize some novel approaches to processing bio-feedstocks with the aim of bypassing the step of a fixed library of ingredients. Two approaches are brought forward: one making use of knowledge-based expert systems and the other making use of applications of metabolic engineering and dynamic combinatorial chemistry.

## Introduction

Global uncertainty over prices of petrochemical feedstocks and the desire to significantly reduce the levels of anthropogenic generation of CO_2_ are the two main drivers behind current rapid development of a replacement supply chain for platform molecules of the chemistry using industries (Perlack et al., 2005; Graham, 2007). What platform molecules produced by new bio-refining technologies would form the basis of the new supply chain is still an issue of a significant debate (Fernando et al., 2006; Smith, 2007; FitzPatrick et al., 2010). This, however, significantly affects downstream technologies, which depend on the catalog of available molecules to develop task-specific products, for example in formulations.

At present the use of bio-feedstocks in product design is relatively limited, due to the small number of molecules available on the market, primarily natural oils, flavor and fragrance substances, nutraceuticals and bio-pharmaceuticals. Very few bio-derived solvents, surfactants or monomers are available at present. However, this range is expected to be rapidly expanded, offering new opportunities for product design. The emerging question is whether our existing methods of product design in formulations and other chemistry-using industries are appropriate for the new developing supply chain based on sustainable renewable feedstocks.

**Figure 1:**
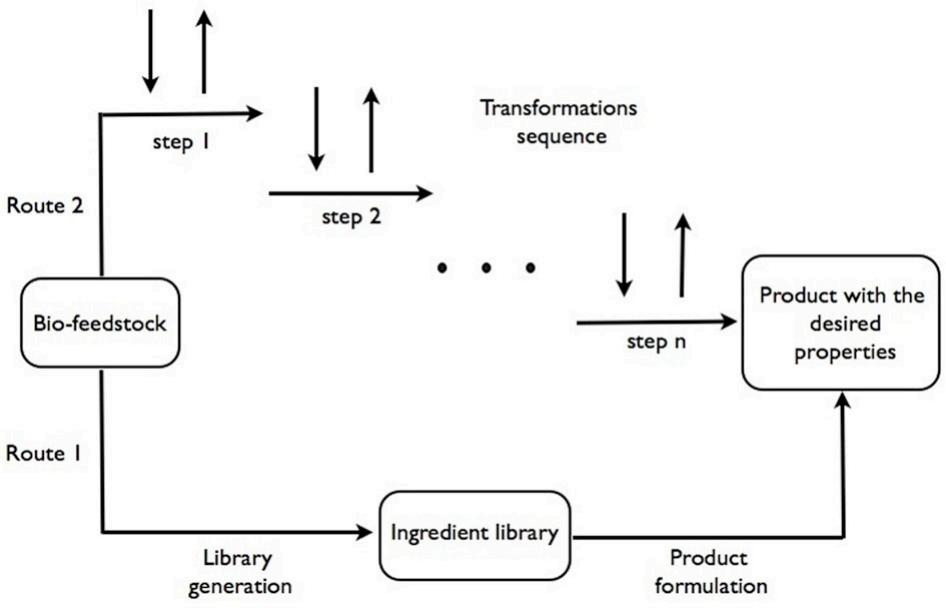
**Schematic representation of both current and proposed approaches to processing bio-feedstocks in product design**.

Our current approach to formulations design is based on heuristic knowledge of formulators that allow to select individual compounds from a library of available materials with known properties, i.e., rheology modifiers, structure-forming agents, color and fragrance substances, bio-actives etc. (Marshall and Alaimo, 2010). The new bio-feedstocks based supply chain will replace some of the usually applied ingredients or offer new ingredients with different functionalities. This is represented by the lower path in Figure 1, from bio-feedstocks to the final products. A significant anticipated difference with the current petrochemicals-derived supply chain of ingredients is in the broader specification of properties of the ingredients due to variability in bio-feedstocks, which may also translate in broader variability in performance of the final products. How acceptable this would be will require further examination.

We speculate that most of the compounds (or functions) that make-up the final product can potentially be obtained from a single, or very few, bio-sources. In this case, there could be an alternative path from feedstocks to products, analogous to a metabolic pathway of a complex organism. This is represented by the top path in Figure 1.

After Corey formalized the concept of retrosynthesis in his seminal work in 1967 (Corey, 1967; Huang et al., 2011) interest very quickly arose in transferring this process away from expert chemists to computer programs written by expert chemists—so called expert systems. Again, Corey was first to develop a program to this end called OCSS (Corey and Wipke, 1969; Moity et al., 2014). Most of these systems were limited by the fact that the rules they were based upon had to be programmed by hand meaning it was virtually impossible to keep up with the rapid development of chemistry (Law et al., 2009). With the advent of databases and advanced computing power this has, however, changed. Millions of chemical reactions performed, and compounds synthesized to date have been systematically recorded and incorporated into a variety of product and reaction databases (Bartosz et al., 2009). The availability of such databases has prompted the development of algorithms and software tools (Jorgensen et al., 1990; Satoh and Funatsu, 1995; Todd, 2005; Socorro and Goodman, 2006; Chen and Baldi, 2009; Law et al., 2009; Nowak and Fic, 2010) to help intelligently explore the gathered information, thus allowing for automatic extraction of new rules for expert systems. Some expert systems are designed to predict major products of a reaction given a combination of starting materials and reagents (Jorgensen et al., 1990; Satoh and Funatsu, 1995; Socorro and Goodman, 2006; Chen and Baldi, 2009), while others are designed to predict, using retrosynthetic analysis, possible starting materials given a target product (Law et al., 2009; Nowak and Fic, 2010).

Alternatively, other approaches to product synthesis are being developed, that do not rely on the information stored in product and reaction databases. These approaches build on advances made in the fields of metabolic engineering (Rozenman et al., 2007; Lee et al., 2011) and dynamic combinatorial chemistry (Hunt and Otto, 2011; Moulin et al., 2012).

The main element of expert systems based approaches is existing chemical knowledge of a large number of compounds and reactions. Recorded in the form of on-line databases, this knowledge is in a format that allows interrogation and rule generation to be performed using expert systems. We speculate that existing chemical knowledge will now include the necessary information for expert systems to, using retrosynthetic analysis, generate (or go some way toward) synthetic routes connecting bio-feedstocks (as starting material) with a number of existing products. Also, as more bio-derived molecules with a variety of different functionalities, are added to the existing supply chain of ingredients, and the corresponding information (the molecules and the starting materials that were used in creating them) is transferred into the existing chemical knowledge, it becomes increasingly possible that synthetic routes connecting bio-feedstocks with new products having desired properties, will be found using expert systems.

Also, new approaches to processing bio-feedstocks in designing products with specific properties through advances made in metabolic engineering and dynamic combinatorial chemistry are envisioned. Within metabolic engineering metabolic pathways are assembled and optimized (by tuning the activity of the intermediate reaction steps) for the production of molecules with desired properties (Yadav and Stephanopoulos, 2010). In dynamic combinatorial chemistry materials are designed as artificial chemical systems that display modulation of functional properties in response to the application of external stimuli (Moulin et al., 2012). These approaches may be viewed as evolution based, as the final product could be arrived at through evolution of the systems involved. For instance, directed evolution is employed in optimizing enzymes and biosynthetic pathways (Leemhuis et al., 2009) involved in synthesis of commercial products (Johannes and Zhao, 2006). Typically, a directed evolution cycle of an enzyme involves: diversification of the parent gene via a chosen method of random mutagenesis and/or *in vitro* gene recombination, mutant enzymes production using the library of mutant genes and identification of improved enzymes whose genes will be used as parents in the next cycle, through a high-throughput screening or a selection method (Zhao et al., 2002). In dynamic combinatorial chemistry, equilibrated libraries of building blocks are generated under reversible conditions and evolve based on an imposed selection process (Corbett et al., 2006). The components of a dynamic combinatorial chemistry experiment are loosely related to components of a system undergoing Darwinian evolution in that a population of individuals (the library), reproduction (based on reversible reactions) and selection (based on binding interactions) are present (Miller, 2009). However, introduction of new diversity into the library of building blocks and iteration of the process are still an issue (Gartner, 2006).

The aim of this paper is to conceptualize approaches to consumer product design that are not reliant on the formulator's experiential knowledge of combining known ingredients into recipes, but, rather, *either* employ expert systems to interrogate the existing chemical knowledge and, thus, extract favorable reaction routes, *or*, by taking advantage of advances made in the fields of metabolic engineering and dynamic combinatorial chemistry, evolve the product generating process until the output product displays the desired properties. Thus, essentially, the novelty of the approaches presented herein lies in the idea of bypassing the step of a fixed ingredient library generation and, hence, avoiding the need for recipe-driven product design.

In Section Methods the proposed ideas are presented. The merits and drawbacks of the proposed ideas are discussed in Section Merits and drawbacks of the proposed approaches and conclusions are drawn in Section Conclusions.

## Methods

### Expert systems based approach

Two variants of one methodology making use of existing chemical knowledge and expert systems are suggested, one aiming for products with known composition and a second targeting known functional properties but with an unknown composition.

#### Methodology A

*Given the target product composition is known, use expert systems and existing chemical knowledge to find an optimal sequence of transformations to perform on bio-feedstocks to arrive at the desired product*.

**Figure 2:**
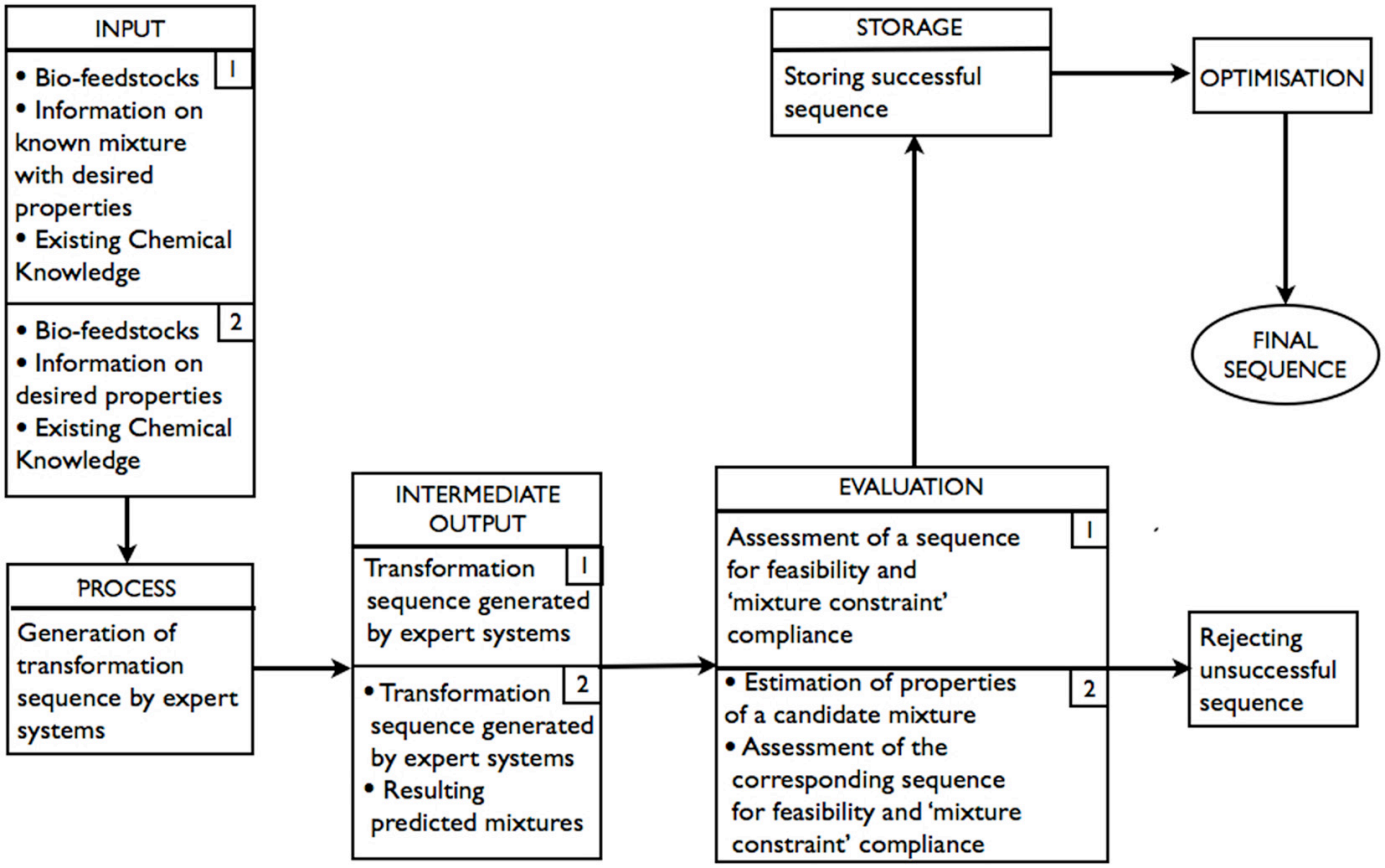
**Schematic representation of the expert systems based approach: route 1–with known product composition; route 2–product composition is unknown**.

As shown in Figure 2 (route 1), bio-feedstocks, the information on composition of the target product/mixture, and existing chemical knowledge are all INPUTs. Generation of transformation sequences is the PROCESS. At this stage, expert systems are used to perform retrosynthetic analysis. INTERMEDIATE OUTPUT is all sequences “deemed” possible (but not necessarily feasible) by the expert system. At the EVALUATION stage, the sequences that are returned as possible are assessed by expert systems for feasibility. As the transformations are to be performed on a mixture of bio-feedstocks, it is important to ensure that, for any reaction in a sequence, the properties of the components that do not take part in the reaction remain unaffected (Paula and Atkins, 2002) (referred to as “mixture constraint” in the diagram). Thus, each feasible sequence is also required to satisfy the “mixture constraint.” It is possible that, after all of the generated sequences have been evaluated, there would be more than one acceptable transformation sequence in STORAGE, in which case, there would be opportunities for OPTIMIZATION.

The idea behind retrosynthesis is to identify an optimal synthesis route connecting a desired product with a commercially available starting material by simplifying the target product through a number of disconnections (the hypothetical reverse of a synthetic step). Each precursor is then in turn examined in the same way until a suitable starting material is identified (Corey, 1991). This can lead to combinatorial explosion with respect to the number of possible reactions to be investigated. For a more detailed discussion the reader is referred to Todd (2005).

One approach to retrosynthetic analysis is the use of generalized reaction rules. These are procedures for evaluating reaction types and are obtained by learning from individual reactions to obtain a generalized scheme for a type of reaction (Gasteiger et al., 2000) through extracting the extended reaction cores of a reaction and then grouping similar reactions together. These groups are in turn generalized to yield a reaction rule (Law et al., 2009). Applying these generalized rules to a molecule allows for the prediction of reactions, or disconnections in this case. As a result new reactions can be discovered (Pham and El-Halwagi, 2012).

A number of systems have recently made use of advanced heuristics and databases to improve the route prediction. In the past retrosynthetic analysis was limited by the requirement for an expert chemist to manually program reaction rules. Law et al. (2009) addressed this problem by using the Beilstein Crossfire database to automatically incorporate all known reactions into the reaction rule generation, while (Christ et al., 2012) reports of efforts at Boehringer Ingelheim to mine electronic laboratory notebooks for reaction rules, extending their reach past the published chemistry. Huang et al. (2011) has sought to address the issue that though a reaction might be feasible on paper it can be very difficult or inefficient to conduct in practice by introducing an accessibility factor allowing for a better differentiation of routes. An alternative approach is the DEF-factor assigned to each transformation by Moity et al. (2014) measuring the sustainability, easiness and scale at which the transformation is used industrially. An interesting future development would be the development of “biocatalytic retrosynthesis” incorporating enzymatic reaction rules (Turner and O'Reilly, 2013). In our opinion many of these recent developments could prove very useful in the planning of synthesis routes for the purpose of formulated product design.

To date, to the best of our knowledge, expert systems based on retrosynthetic analysis are yet to be employed in development of consumer products. However, the capabilities of such expert systems have been validated in specific cases. For instance, in Law et al. (2009), Route Designer expert system was able to find a synthetic transformation sequence for Zatosetron, a potent, selective and long acting 5HT receptor antagonist used in the treatment of nausea and emesis associated with oncolytic drugs. First, rule extraction, using MOS reaction database from Accelrys and the Beilstein Crossfire reaction database from Elsevier, was performed. The program was then presented with the target and a database of 120 k of starting materials. The search for possible synthetic sequences followed. The assessment of feasibility of suggested sequences is inbuilt in the algorithm and was performed at a transformation level as the sequences were assembled. A number of ranked possible, feasible sequences were then presented as the output.

An alternative approach to the same end would be the use of the Network of Organic Chemistry (NOC). In 1990 (Lawson and Kallies, 1990) observed that the Beilstein database forms an implicit network resulting in “a map of practically all known synthetic pathways from almost any starting material to almost any product.” In 2005 this idea was further developed by Fialkowski et al. (2005) who first converted the Beilstein database into a network, called the NOC, and further investigated various properties of the NOC in a series of publications (Fialkowski et al., 2005; Bishop et al., 2006; Grzybowski et al., 2009; Gothard et al., 2012; Kowalik et al., 2012; Soh et al., 2012). The important feature of this network is that a computer with an effective network search algorithm is able to optimize a synthesis route very quickly. Providing that the required reactions have been published this can be done without having to take recourse to modeling or lengthy literature research. This would be a vast improvement in efficiency for the design of synthesis routes compared to the status quo.

Though this methodology is interesting in its own right it would be desirable to extend it to the prediction of unknown reactions not contained in the network. To this end a number of approaches could be used. In statistical terminology the problem of discovering reaction not presently contained in the network equates to an edge prediction problem. One purely statistical, though potentially very powerful, approach would be the use of hierarchical networks, for a good discussion of which the reader is referred to Barabási and Oltvai (2004). The potential of this approach, as well as a suitable algorithm, has been demonstrated in (e.g., Clauset et al., 2008). This assumes that the NOC exhibits hierarchical behavior which would need to be investigated (for details of which reference is made to Ravasz et al. (2002) though other statistical techniques for the purpose of edge-prediction could be used should this not be the case (Lü and Zhou, 2011). A different approach that would make more use of chemical intuition would be to run a search in the NOC in a generalized format such as the extended reaction core methodology used by Law et al. (2009) or other reaction rule generation methodologies. This would return a list of all similar reactions from which it might be possible to extrapolate as to how the unknown reaction currently under investigation could be carried out.

A question equally as important as the discovery of new reactions is that of making reactions already existing within the NOC more efficient by using better catalysts. For this purpose the previously described extended reaction core approach could be used. It would however also be desirable to conduct computational screening of catalysts. To this end reference is made to Nørskov et al. (2011). This paper demonstrates the possibility of reducing a DFT simulation to two descriptors and several scaling relationships for the other variables, which allows for a descriptor-based search for new catalysts potentially making the process far more efficient.

#### Methodology B

*Given only the desired properties of the product are known, use expert systems and existing chemical knowledge to perform a series of transformations on bio-feedstocks to arrive at a product with the desired set of properties*.

As shown in Figure 2 (route 2), a mixture of bio-feedstocks and the information on the product's properties are INPUTs. Generation of possible transformation sequences is, again, the PROCESS and involves the use of expert systems to perform forward synthesis. INTERMEDIATE OUTPUT is all sequences and the corresponding predicted resulting products “deemed” possible (but not necessarily feasible) by the expert system. At the EVALUATION stage, the sequences that are returned as possible are assessed by expert systems for feasibility. The properties of predicted resulting products of feasible sequences are estimated. The sequence is accepted if the estimates of properties are within the tolerance required and the “mixture constraint” is not violated. Again, it is possible that there would be more than one acceptable transformation sequence, in which case, there would be an opportunity for optimization.

At present, within the context of the above-mentioned approach, expert systems are able to assist with generation and evaluation (in terms of feasibility only) of the candidate products. In Chen and Baldi (2009), for instance, examples are given of an expert system, with reaction predicting capabilities, being used to generate products in two ways: generating products systematically, given a number of predetermined starting materials, and generating products that are similar to a given target product using the precursors of the target product as starting materials. Also, the design of the expert system ensures that it only predicts synthetically feasible products. In Moity et al. (2014), a computer-assisted tool named GRASS is presented that, given a chosen bio-based building block (molecule/s obtained through chemical or biochemical transformation of a biomass feedstock) and co-reactants, generates a library of possible products that are then assessed for synthesis feasibility and application-specific properties. The authors demonstrate the capabilities of the GRASS program by virtually designing agro-based solvents known in the literature. The scheme in full, as in Figure 2 (route 2), has not been attempted, to the best of our knowledge.

### Evolution-based approach

**Figure 3:**
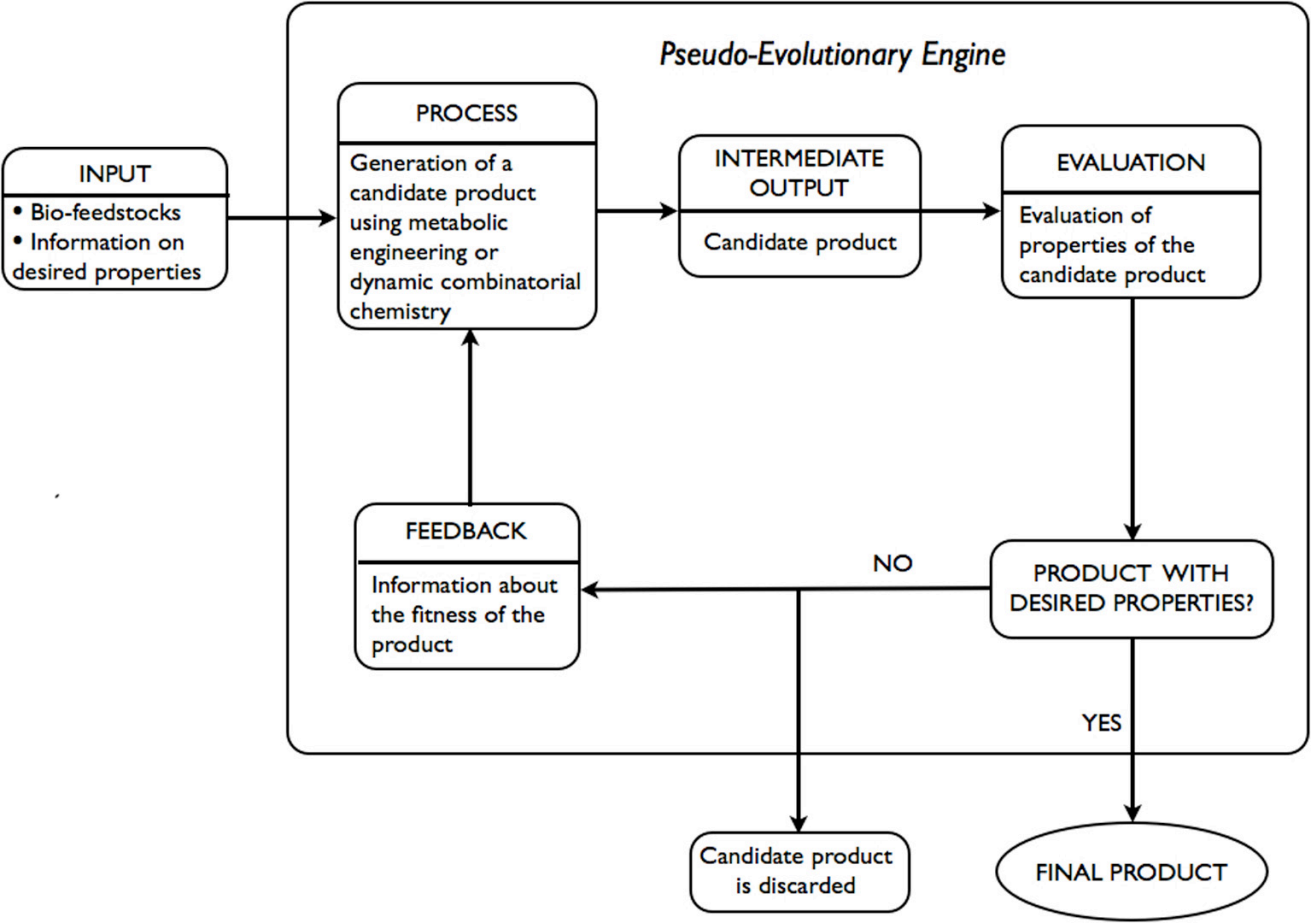
**Schematic representation of evolution-based approach**.

An alternative to expert systems approach involves the use of ideas developed in the fields of metabolic engineering and dynamic combinatorial chemistry. Given only the desired properties of the product are known, a pseudo evolutionary engine is used, as illustrated in Figure 3, to arrive at a product with the desired properties. INPUT includes bio-feedstocks and information on desired properties of a product. At the PROCESS stage an INTERMEDIATE OUTPUT-candidate product is generated via the application of metabolic engineering or dynamic combinatorial chemistry. At the EVALUATION stage an assessment of the candidate product, in terms of whether the estimates of desired properties are within required tolerance or not, is performed. If the candidate product does not meet the requirements, it is discarded. Using FEEDBACK, adjustments to the generation step are made and a new candidate product is generated. The process is repeated until the desired properties are within the tolerance required.

The majority of components of the above-mentioned approach are in existence. Within metabolic engineering, a number of strains are often designed (PROCESS, INTERMEDIATE OUTPUT) and evaluated in terms of percentage yield of the desired product (EVALUATION). Following the evaluation, adjustments are made and often involve deletion of unnecessary or optimization of necessary genes or a combination thereof. Within dynamic combinatorial chemistry, dynamic combinatorial libraries (DCLs) are constructed (PROCESS) and evaluated in terms of functional modularity in response to an external stimuli (INTERMEDIATE OUTPUT, EVALUATION). Following the evaluation, adjustments to the construction of DCLs are made and often involve: replacement of some of the constituents within DCLs, alteration of the reversible chemistry used, a change in the external stimuli applied. For instance, in Hanai et al. (2007) authors report the use of metabolic engineering in designing a synthetic pathway in *E. coli* for the production of isopropanol (Papa, 2005), a secondary alcohol that is used, among other ways, in pharmaceutical applications. The successful strain was developed through expression of a variety of combinations of genes from a selected list of known strains. In Jung et al. (2010) authors give account of the use of metabolic engineering and enzyme engineering in development of *E. coli* strains for the production of biomass-derived plastics, polylactic acid, and its copolymers. The techniques applied in optimizing metabolic pathways of the organism were deletion of unnecessary genes and optimization of the expression of necessary genes based on *in silico* genome-scale flux analysis combined with rational approach.

In Nasr et al. (2009a,b), Gareiss et al. (2008), Bugaut et al. (2008) the authors make use of dynamic combinatorial chemistry in identification of enzyme-inhibitors. Commonly, the steps involved include generation of DCLs under thermodynamic control using a predetermined type of reversible chemistry and assessment of interactions between constituents of the libraries and the target through “measuring” of the change in the composition of a DCL upon introduction of a target.

## Merits and drawbacks of the proposed approaches

Method A of the expert systems based approach is, perhaps, the easiest to implement. As the target product is known from the start, this methodology does not involve property estimation, which can be difficult to do analytically and is costly. Retrosynthetic analysis, employed as part of this methodology, can also result in the discovery of new reactions. Its main drawback is the fact that a transformation sequence, connecting given starting materials with a target product, might simply not exist, as some or all of the required chemistry may not have been carried out yet.

Method B of the expert systems based approach, however, although also dependent on a large variety of chemistry to have been done, is not constrained by the necessity of finding a transformation sequence to a given target product, but rather by a set of properties that the target product should exhibit, which somewhat liberates the search. In fact, the final output of this approach may, intriguingly, be a product that has not been considered before. The main foreseen difficulty with this methodology is the costs associated with the necessity for property estimation, either analytically or through an experiment, for each candidate product.

The main attraction of the evolution-based approach is the potential for discovery of novel products with desired properties and the potential to discover new knowledge. The biomimetic approach of evolution-based process development requires implementation of generic principles of evolutionary development, which will necessarily sample a very large space of potential process variants. This methodology depends on the ability to sample the outcomes of each evolutionary step and to make adequate decisions, both, about the new, yet unknown phenomena that took place and which could potentially be exploited, as well as about the following steps in the process evolution. As in natural evolution, the approach is not blind, but follows some generic rules. The envisioned evolutionary approach would, at the very basic level, involve an “adjustment” (mutation) step, applied iteratively, to evolve a product generating process. However, it is not unreasonable to think of the possibility of evolving a population of product generating processes, in which case selection and crossover steps would come into play. To allow the approach to converge on the optimal (near optimal) process (or population of processes) within the allocated amount of resources and/or time, adequate selection, crossover and mutation operators would need to be designed.

The evolution-based approach has the potential to not only discover novel products with desired properties, but, intriguingly, products with additional, perhaps unexpected, functions/properties. These (additional functions/properties), of course, can be undesirable and the candidate product discarded, in the context of the product sought. However, the new knowledge, thus acquired, may benefit the design of new products and, hence, should be retained. In addition to the potential to facilitate development of other products with different functionality, the data collected using the evolution-based approach could be utilized to build physical/empirical models of the underlying physical processes involved in product generation (or help improve the existing methodology).

## Conclusions

In this short investigation new methods of processing bio-feedstocks in consumer product design were discussed. An attempt was made to conceptualize some novel approaches to processing bio-feedstocks with the aim of bypassing the step of a fixed library of ingredients. Two approaches were brought forward and discussed: one making use of expert systems and the other, evolution-based approach, making use of advances made in the fields of metabolic engineering and dynamic combinatorial chemistry. The two main components of both approaches are: generation of a number of candidate transformation sequences/process variants and properties estimation of the candidate products (second variant of the expert systems based approach and the evolution based approach). Both [components] present challenges. *In silico* generation of candidate products/transformation sequences using expert systems, given sufficient information is contained within existing chemical knowledge, is very time and material efficient, however, property estimation of the candidate products (second variant of the expert systems based approach) is likely to be material-intensive and time-consuming. By contrast, generation of candidate products using the evolution- based approach involves the design and set up of experiments, which may require a substantial investment of time and resources. However, this initial investment would pay off at the property estimation stage. It is possible that the problem of identifying the transformations needed in processing bio-feedstocks could be solved through the use of both approaches, as some transformations may only be done via an expert systems based or evolution-based approach.

### Conflict of interest statement

The authors declare that the research was conducted in the absence of any commercial or financial relationships that could be construed as a potential conflict of interest.
